# Validation and derivation of short-term prognostic risk score in acute decompensated heart failure in China

**DOI:** 10.1186/s12872-022-02743-1

**Published:** 2022-07-07

**Authors:** Hong-Liang Zhao, Xiao-Li Gao, Ying-Hua Liu, Sen-Lin Li, Qi Zhang, Wei-Chao Shan, Qun Zheng, Jiang Zhou, Yong-Zheng Liu, Li Liu, Nan Guo, Hong-Sen Tian, Qing-Min Wei, Xi-Tian Hu, Ying-Kai Cui, Xue Geng, Qian Wang, Wei Cui

**Affiliations:** 1 First Division, Department of Cardiology, The Second Hospital of Hebei Medical University, Heping West Road No. 215, Shijiazhuang, 050000 Hebei province China; 2 Department of Cardiology, The First Hospital of Hebei Medical University, Shijiazhuang, 050031 Hebei province China; 3 Department of Cardiology, Huabei Petroleum Administration Bureau General Hospital, Renqiu, 062552 Hebei Province China; 4 Department of Cardiology, Zhangjiakou First Hospital, Zhangjiakou, 075000 Hebei Province China; 5 Department of Cardiology, Baoding First Central Hospital, Baoding, 071000 Hebei Province China; 6 Department of Cardiology, Affiliated Hospital of Chengde Medical University, Chengde, 067000 Hebei Province China; 7 Department of Cardiology, Hengshui People’s Hospital, Hengshui, 053000 Hebei Province China; 8 Department of Cardiology, Chengde Central Hospital, Chengde, 067024 Hebei Province China; 9 Department of Cardiology, Qinhuangdao First Hospital, Qinhuangdao, 066099 Hebei Province China; 10 Department of Cardiology, Cangzhou Central Hospital, Cangzhou, 061011 Hebei Province China; 11 Department of Cardiology, Handan Central Hospital, Handan, 056000 Hebei Province China; 12 Department of Cardiology, Xingtai People’s Hospital, Xingtai, 054001 Hebei Province China; 13 Department of Cardiology, Shijiazhuang People’s Hospital, Shijiazhuang, 050011 Hebei Province China; 14 Department of Cardiology, The 252nd Hospital of People’s Liberation Army, Baoding, 071000 Hebei Province China

**Keywords:** Acute decompensated heart failure, Prognosis, Risk score, Short-term

## Abstract

**Background:**

Few prognostic risk scores (PRSs) have been routinely used in acute decompensated heart failure (ADHF). We, therefore, externally validated three published PRSs (3A3B, AHEAD, and OPTIME-CHF) and derived a new PRS to predict the short-term prognosis in ADHF.

**Methods:**

A total of 4550 patients from the Heb-ADHF registry in China were randomly divided into the derivation and validation cohorts (3:2). Discrimination of each PRS was assessed by the area under the receiver operating characteristic curve (AUROC). Logistic regression was exploited to select the predictors and create the new PRS. The Hosmer–Lemeshow goodness-of-fit test was used to assess the calibration of the new PRS.

**Results:**

The AUROCs of the 3A3B, AHEAD, and OPTIME-CHF score in the derivation cohort were 0.55 (95% CI 0.53–0.57), 0.54 (95% CI 0.53–0.56), and 0.56 (95% CI 0.54–0.57), respectively. After logistic regression analysis, the new PRS computed as 1 × (diastolic blood pressure < 80 mmHg) + 2 × (lymphocyte > 1.11 × 10^9^/L) + 1 × (creatinine > 80 μmol/L) + 2 × (blood urea nitrogen > 21 mg/dL) + 1 × [BNP 500 to < 1500 pg/mL (NT-proBNP 2500 to < 7500 pg/mL)] or 3 × [BNP ≥ 1500 (NT-proBNP ≥ 7500) pg/mL] + 3 × (QRS fraction of electrocardiogram < 55%) + 4 × (ACEI/ARB not used) + 1 × (rhBNP used), with a better AUROC of 0.67 (95% CI 0.64–0.70) and a good calibration (Hosmer–Lemeshow *χ*^2^ = 3.366, *P* = 0.186). The results in validation cohort verified these findings.

**Conclusions:**

The short-term prognostic values of 3A3B, AHEAD, and OPTIME-CHF score in ADHF patients were all poor, while the new PRS exhibited potential predictive ability. We demonstrated the QRS fraction of electrocardiogram as a novel predictor for the short-term outcomes of ADHF for the first time. Our findings might help to recognize high-risk ADHF patients.

## Background

Acute decompensated heart failure (ADHF) is a prevalent clinical syndrome with a poor prognosis in internal medicine and emergency department, and the majority of ADHF patients require hospitalization for further treatment [1, 2]. Despite remarkable therapeutic advancements, the mortality and rehospitalization rates of ADHF remain high [3], most conspicuously during hospitalization and the early post-discharge period [4–6], dubbed “the vulnerable phase” for heart failure (HF) [7]. Data from China show that ADHF is the leading cause of hospitalization in patients > 65 years old, with hospitalization mortality of 3% and an around 50% short-term readmission rate [8].

It is critical to accurately predict prognosis in patients with ADHF during and after hospitalization to optimize treatment decisions, thereby reducing readmissions and deaths [9]. For these purposes, several prognostic risk scores (PRSs) in patients with HF have been developed [10–13]. However, most of these scores have not been thoroughly externally validated and the effective values of predictors are differ among races and regions [14, 15]. Moreover, current scoring systems paid less attention to outcomes in patients with ADHF during the vulnerable phase, and it is unclear whether they can be directly applied to ADHF patients.

Among the previously published PRSs, the 3A3B score is developed for the Japanese HF preserved left ventricular ejection fraction (LVEF) (HFpEF). From a total of 14 covariates, age, albumin, anemia, BMI, BNP or NT-proBNP, and BUN were selected as long-term prognostic variables [13]. The predictive value was confirmed in the external validation in Asian cohorts. The discrimination abilities were all excellent in both derivation and validation cohorts (c-index = 0.708). However, the accuracy of this score in predicting short-term prognosis in ADHF patients is uncertain.

The AHEAD score is a simple tool based on 5 comorbidities (Atrial fibrillation, Hemoglobin (anemia), Elderly, Abnormal renal parameters (creatinine), and Diabetes mellitus) used to predict the short and long term prognosis of hospitalized patients with acute HF (AHF) in the European population. It was derived from a prospective multicenter registry. The score was externally validated in the GREAT registry consisting of nine AHF cohorts from Italy, Spain, France, Argentina, Finland, Switzerland, the USA, Tunisia, and Austria [12]. Nevertheless, its prognostic efficacy in Asian patients with ADHF has not been proven.

The OPTIME-CHF score has been suggested as a way to predict the risk of 60-day mortality for hospitalized patients with decompensated HF. Using data from a cohort from the United States, 5 routine variables on admission, including age, SBP, sodium, New York Heart Association (NYHA) class IV, and BUN, were found to be independently associated with the prognosis. The discrimination of the current score was excellent (c-index = 0.77). The authors further developed a nomogram based on each factor for bedside application [10], which, however, was not externally validated.

Considering the research context (population and regions), as well as the availability of variables, we selected the 3A3B [13], AHEAD [12], and OPTIME-CHF [10] scores as the alternative PRSs. This study therefore aimed to validate whether the 3A3B, AHEAD, and OPTIME-CHF could accurately predict the composite of in-hospital all-cause mortality, 30-day all-cause readmission, or 30-day all-cause mortality after discharge in ADHF patients. Meanwhile, we derived a new PRS and compared it with the existing systems. We believe this will contribute to the prognostic risk assessment of ADHF patients.

## Methods

### Study population

The study population consisted of hospitalized patients with ADHF enrolled in the Hebei Acute Decompensated Heart Failure (Heb-ADHF) registry (ChiCTR-POC-17014020) database, a prospective, multicenter, open study designed in real-world to assess risk predictors of influencing comprehensive treatment and short-term prognosis in patients with ADHF. From March 2016 and December 2018, patients were consecutively recruited in 13 tertiary hospitals in Hebei Province, China.

ADHF was defined as new-onset AHF or decompensation of chronic HF (CHF) [16]. All of the patients in this study were hospitalized with ADHF. Patients were eligible for inclusion if they were: (1) age ≥ 18 years; (2) unplanned admission; (3) with typical ADHF symptoms or signs; and (4) brain natriuretic peptide (BNP) > 100 pg/mL or N-terminal pro-brain natriuretic peptide (NT-proBNP) > 300 pg/mL. Diagnosis and treatment of ADHF were determined by the cardiologist-physicians according to the clinical guideline [8]. Exclusion criteria were: (1) hospital stay < 24 h (refuse further treatment or request a referral to higher-level hospitals); (2) heart transplantation; (3) renal replacement therapy; (4) massive stroke; (5) concomitant terminal disease; or (6) patients lost to follow-up. All eligible subjects were randomly divided into the derivation cohort and the validation cohort at a ratio of 3:2. The derivation cohort was used to validate the published PRSs and establish the new model, whereas the validation cohort was used to validate the findings.

### Data collection

We recorded the following clinical data on admission of each enrolled patient: (1) demographic characteristics; (2) clinical history; (3) physical examination; (4) laboratory tests; (5) chest radiography (6) electrocardiogram; (7) echocardiography; and (8) medical treatment. Variables analysis of the outcome events group revealed the following cut-offs: age, 65 year-old; body mass index (BMI), 24 kg/m^2^; length of hospital stay (LoHS), 10 days; systolic blood pressure (SBP), 130 mmHg; diastolic blood pressure (DBP), 80 mmHg; anemia, < 13.0 g/dL in men or < 12.0 g/dL in women; red blood cells (RBC), 4.15 × 10^12^/L; neutrophil, 4.37 × 10^9^/L; lymphocyte, 1.11 × 10^9^/L; platelet, 155 × 10^9^/L; total cholesterol (TC) 3.6 mmol/L; low density lipoprotein cholesterol (LDL-C), 2.0 mmol/L; creatinine, 80 μmol/L; blood urea nitrogen (BUN), 21 mg/dL; aspartate aminotransferase (AST), 32 U/L; alanine aminotransferase (ALT), 59 U/L; QRS fraction, 55%; left ventricular ejection fraction (LVEF), 36%; left atrial diameter (LAD), 41 mm. Phase values were used for BNP (NT-proBNP), 100 to < 500 (300 to < 2500), 500 to < 1500 (2500 to < 7500), and ≥ 1500 (≥ 7500) pg/mL, respectively. QRS fraction is defined as the sum of the R-wave amplitudes of the standard 12 leads (ΣR) divided by the sum of the absolute values of the QRS wave amplitudes of the 12 leads (ΣQRS) in electrocardiogram, i.e. (ΣR/ ΣQRS) × 100%. All cut-offs were calculated based on the predictive power of each variable for the outcome event.

### Study endpoint and follow-up

The primary endpoint was the composite of in-hospital all-cause mortality, 30-day all-cause readmission, or 30-day all-cause mortality after discharge. The follow-up programs for all patients were carried out according to the original plan of the Heb-ADHF study via medical records, physician’s office visits, or telephone interviews from admission to 30 days after discharge.

### Statistical analysis

Continuous variables are presented as means ± standard deviations or medians with interquartile range whenever appropriate; categorical variables are presented as frequencies (*n*) and proportions (%). The few missing data are replaced by the expectation–maximization method. The Student *t*-test or Mann–Whitney *U* test was used to evaluate differences between groups for continuous variables and the Chi-square (*χ*^2^) test for categorical variables whenever appropriate. The discrimination of each PRS was evaluated by the area under the receiver operating characteristic curve (AUROC) with a 95% confidence interval (CI) to assess ability to predict the endpoint. To begin, univariate logistic regression analyses were used to identify risk factors associated with outcome events. Variables with *P*-values of < 0.2 in univariate analyses were entered into the multivariable analysis. Then, variables with *P*-values of < 0.05 in the multivariate logistic regression were considered as independent variables for the new PRS. Odds ratios (ORs) with 95% CIs were calculated for the logistic regression analysis. The relative significance of each predictor within the final PRS model was assessed by the value of the partial Wald *χ*^2^ statistic minus the degrees of freedom (*df*) of predictors (*χ*^2^-df). In the new PRS model, we assigned the scores (points) for each independent variables based on their value of (*χ*^2^-*df*): (*χ*^2^-*df*) ≤ 5.0 assigned as 1 point, 5.0 < (*χ*^2^-*df*) ≤ 10 assigned as 2 points, 10 < (*χ*^2^-*df*) ≤ 15 assigned as 3 points, and (*χ*^2^–*df*) > 15 as 4 points. The *Hosmer–Lemeshow* goodness-of-fit test was used to assess calibration. Using the *Z* test, we also compared the predictive accuracy of new PRS with that of the 3 previously published PRS by AUROCs. The correlation between different score points and different outcome events was analyzed by *Mantel–Haenszel* trend test and *Pearson* correlation test. Two-sided *P* < 0.05 was deemed statistically significant. Statistical analyses were performed using SPSS software (version 26.0) and the AUROC curves were conducted using the Medcalc software (version 20.0.3).

## Results

### Population characteristics

Patients including 43 discharged within 24 h, 1 with heart transplant, 11 with renal replacement, 2 with massive stroke, 4 with malignant tumors, and 135 lost to follow-up were excluded. Finally, a total of 4550 patients from the Heb-ADHF registry were enrolled in this study, who were randomly divided into the derivation cohort (2,745) and the validation cohort (1,805) at a ratio of 3:2. The incidence of the composite of outcomes was 13.9% (381/2745) in the derivation and 13.9% (249/1805) in the validation cohorts. For both cohorts, the age, male, BMI, smoking, drinking, HF duration, number of hospitalizations for heart failure (NH-HF), LoHS, SBP, DBP, heart rate, etiology, heart function, comorbidities, urine protein, laboratory blood tests, chest radiography, electrocardiogram, echocardiography, medical treatment, and risk of outcome events were comparable without significant difference (*P* > 0.05). The clinical characteristics of derivation and validation cohorts are summarized in Table 1.

**Table 1:** Comparison of clinical characteristics between the derivation and validation cohorts

Characteristic	Derivation cohort (*n* = 2745)	Validation cohort (*n* = 1805)	*P*-Value
Age, years	67.8 ± 12.9	67.2 ± 13.0	0.126
Male, *n* (%)	1603 (58.4)	1057 (58.6)	0.913
BMI, kg/m^2^	24.5 ± 4.5	24.5 ± 3.9	1.000
Smoking, *n* (%)	651 (23.7)	411 (22.8)	0.461
Drinking, *n* (%)	575 (20.9)	347 (19.2)	0.157
LoHS, days	10.9 ± 5.8	11.2 ± 6.9	0.114
SBP, mmHg	132.6 ± 24.4	132.7 ± 25.0	0.893
DBP, mmHg	80.4 ± 15.5	80.7 ± 16.2	0.530
Heart rate, times/min	87.8 ± 23.5	88.4 ± 23.7	0.401
Etiology: ischemic, *n* (%)	1499 (54.6)	957 (53.0)	0.293
Heart function, *n* (%)			
NYHA class IV	1257 (45.8)	836 (46.3)	0.729
Killip class IV	40 (1.5)	39 (2.2)	0.076
Comorbidities, *n* (%)			
Atrial fibrillation	870 (31.7)	549 (30.4)	0.362
Hypertension	1580 (57.6)	1068 (59.2)	0.281
Coronary artery disease	1376 (50.1)	860 (47.6)	0.101
Diabetes mellitus	706 (25.7)	493 (27.3)	0.233
Chronic kidney disease	189 (6.9)	111 (6.1)	0.328
Stroke	501 (18.3)	299 (16.6)	0.144
Urine protein + , *n* (%)	315 (11.5)	243 (13.5)	0.046
Blood findings			
Red blood cells, 10^12^/L	4.30 (3.86, 4.69)	4.29 (3.82, 4.70)	0.868
Hemoglobin, g/L	130.3 ± 22.8	130.3 ± 23.2	1.000
Hematocrit, %	39.11 ± 8.94	39.32 ± 8.36	0.427
white blood cells, 10^9^/L	7.10 (5.69, 9.04)	7.10 (5.64, 9.10)	0.883
Neutrophil, 10^9^/L	4.83 (3.69, 6.62)	4.81 (3.60, 6.70)	0.875
Lymphocyte, 10^9^/L	1.40 (1.00, 1.82)	1.39 (1.00, 1.84)	0.696
Platelet, 10^9^/L	200.6 ± 70.4	201.9 ± 71.8	0.545
Glucose, mmol/L	5.80 (4.91, 7.34)	5.80 (4.94, 7.28)	0.954
Creatinine, μmol/L	82.0 (66.3, 103.0)	83.1 (67.0, 106.0)	0.135
BUN, mg/dL	20.5 (15.5, 29.2)	20.5 (15.8, 30.4)	0.240
Na^+^, mEq/L	138.9 ± 4.9	139.2 ± 4.5	0.037
Total cholesterol, mmol/L	3.96 (3.30, 4.69)	3.92 (3.30, 4.62)	0.564
LDL-C, mmol/L	2.42 ± 0.88	2.33 (1.84, 2.91)	0.592
ALT, U/L	24.0 (15.0, 40.4)	24.1 (15.0, 42.0)	0.546
AST, U/L	25.0 (18.5, 41.0)	25.4 (19.0, 42.1)	0.184
Albumin, g/L	38.28 ± 5.35	38.48 ± 5.37	0.218
BNP, pg/mL^b^	797.0 (400.2, 1540.0)	855.5 (382.5, 1620.0)	0.451
NT-proBNP, pg/mL^c^	4644.9 (2408.6, 8794.6)	4893.5 (2351.0, 9189.3)	0.663
cTnI/T (higher than normal), *n* (%)			
1 to < 3 times	149 (5.4)	94 (5.2)	0.746
≥ 3 times	556 (20.3)	380 (21.1)	0.515
Electrocardiogram, *n* (%)			
Ventricular premature beat	236 (8.6)	153 (8.5)	0.886
Left bundle branch block	165 (6.0)	102 (5.7)	0.613
QRS fraction, % ^a^	54.24 ± 13.48	53.82 ± 14.00	0.311
SPC (X-ray), *n* (%)			
pulmonary congestion	708 (25.8)	445 (24.7)	0.388
Pulmonary edema	670 (24.4)	451 (25.0)	0.658
Cardiothoracic ratio > 50%, *n* (%)	1285 (46.8)	810 (44.9)	0.200
Echocardiogram			
LVEF, %	45.2 ± 12.7	45.2 ± 12.9	1.000
Left atrial diameter, mm	43.2 ± 9.0	43.3 ± 8.7	0.710
LVEDD, mm	55.0 (48.1, 62.7)	55.0 (49.0, 62.0)	0.794
Medical therapy, *n* (%)			
Loop diuretics	2489 (90.7)	1618 (89.6)	0.250
ARA	2331 (84.9)	1506 (83.4)	0.178
Hydrochlorothiazide	248 (9.0)	164 (9.1)	0.953
Tolvaptan	35 (1.3)	20 (1.1)	0.614
β-blocker	1916 (69.8)	1268 (70.2)	0.746
ACEI/ ARB	1595 (58.1)	1005 (55.7)	0.106
Calcium channel blockers	422 (15.4)	260 (14.4)	0.370
Nitrates	1339 (48.8)	902 (50.0)	0.431
Digitalis	863 (31.4)	541 (30.0)	0.295
rh-BNP	235 (8.6)	183 (10.1)	0.072
Levosimendan	130 (4.7)	88 (4.9)	0.829
Antiplatelets	1830 (66.7)	1184 (65.6)	0.455
Statin	1831 (66.7)	1172 (64.9)	0.217
Anticoagulants	338 (12.3)	238 (13.2)	0.387
Outcome events, *n* (%)	381 (13.9)	249 (13.9)	1.000
all-cause in-hospital mortality	130 (4.7)	68 (3.8)	
all-cause 30-day readmission	210 (7.7)	149 (8.3)	
all-cause 30-day mortality after discharge	41 (1.5)	32 (1.8)	

*BMI* body mass index, *TH-HF* times of hospitalizations for heart failure, *LoHS* length of hospital stay, *SBP* systolic blood pressure, *DBP* diastolic blood pressure, *BUN* blood urea nitrogen, *LDL-C* low density lipoprotein cholesterol, *ALT* alanine aminotransferase, *AST* aspartate aminotransferase, *BNP* brain natriuretic peptide, *NT-proBNP* N-terminal pro-brain natriuretic peptide, *cTnI/T* cardiac troponin I/T, *SPC* signs of pulmonary congestion, *LVEF* left ventricular ejection fraction, *NYHA* New York Heart Association, *LVEF* left ventricular ejection fraction, *LVEDD* left ventricular end-diastolic diameter, *ARA* aldosterone receptor antagonists, *ACEI* angiotensin converting enzyme inhibitor, *ARB* angiotensin receptor blocker, *rh-BNP* recombinant human brain natriuretic peptide

^a^QRS fraction is calculated by sum of the R-wave amplitudes of the standard 12 leads (ΣR) and dividing by the sum of the absolute values of the QRS wave amplitudes of the 12 leads (ΣQRS), i.e. (ΣR/ ΣQRS) × 100%

^b^The data of BNP were available in 2,916 patients, including 1,796 in derivation cohort and 1,120 in validation cohort

^c^The data of NT-proBNP were available in 1638 patients, including 950 patients in derivation cohort and 688 in validation cohorts

### Validation of previous scores in the derivation cohort

The results showed that the discriminations of 3A3B, AHEAD, and OPTIME-CHF score were all poor on the composite of in-hospital all-cause mortality, 30-day all-cause readmission, or 30-day all-cause mortality after discharge in the derivation cohort, with AUROCs of 0.55 (95% CI 0.53–0.57), 0.54 (95% CI 0.53–0.56), and 0.56 (95% CI 0.54–0.57), respectively. Only the specificity of 3A3B score was acceptable (85.3%), but the sensitivity was unsatisfactory (22.0%).

### Logistic regression analysis and the new risk score model

Logistic regression analysis was performed in the derivation cohort (Table 2). Following univariate analysis, 27 variables (*P* < 0.2) entered into the multivariable analysis: sex (male), BMI, LoHS, DBP, anemia, RBC, neutrophil, lymphocyte, platelet, TC, LDL-C, creatinine, BUN, AST, ALT, cTnI/T, BNP (NT-proBNP), QRS fraction, pulmonary congestion (X-ray), LVEF, LAD, ACEI/ARB, β-blocker, Tolvaptan, recombinant human brain natriuretic peptide (rhBNP), calcium channel blockers, and Statin. However, multivariate analysis revealed that only 8 variables (*P* < 0.05) [DBP, lymphocyte, creatinine, BUN, BNP (NT-proBNP), QRS fraction of electrocardiogram, ACEI/ARB, and rhBNP] maintained an independent correlation with the composite endpoint, which were included into the development of the final model.

**Table 2:** Logistic regression analysis of the composite outcomes in the derivation cohort

Variables	Univariate analysis		Multivariate analysis		Model selection	
	OR (95% CI)	*P–Value*	OR (95% CI)	*P-Value*	*χ*^2^-df	Score
Age ≥ 65 years	0.916 (0.734–1.144)	0.440				
Sex (meal)	1.183 (0.951–1.470)	0.131	1.074 (0.851–1.356)	0.546		
BMI < 24 kg/m^2^	1.160 (0.934–1.442)	0.180	1.061 (0.842–1.338)	0.615		
LoHS ≥ 10 days	1.256 (1.007–1.567)	0.043	1.206 (0.960–1.517)	0.108		
SBP < 130 mmHg	1.150 (0.926–1.428)	0.206				
DBP < 80 mmHg	1.264 (1.017–1.570)	0.034	1.286 (1.021–1.619)	0.032	4.58	1
NYHA (Killip) class IV	1.000 (0.805–1.242)	0.998				
Diabetes mellitus	1.032 (0.807–1.321)	0.800				
Anemia	1.188 (0.953–1.481)	0.125	0.988 (0.718–1.361)	0.943		
Red blood cells ≤ 4.15 × 10^12^/L	1.278 (1.028,1.588)	0.027	0.653 (0.184–2.315)	0.510		
Neutrophil > 4.37 × 10^9^/L	1.301 (1.046–1.617)	0.018	2.013 (0.562–7.214)	0.283		
Lymphocyte > 1.11 × 10^9^/L	1.832 (1.423–2.358)	< 0.001	1.738 (1.220–2.475)	0.002	9.37	2
Platelet ≥ 155 × 10^9^/L	1.305 (1.004–1.696)	0.046	1.282 (0.967–1.700)	0.084		
Total cholesterol < 3.6 mmol/L	1.429 (1.147–1.781)	0.001	1.362 (0.981–1.890)	0.065		
LDL–C < 2.0 mmol/L	1.319 (1.054–1.650)	0.015	1.103 (0.790–1.541)	0.564		
Creatinine > 80 μmol/L	1.386 (1.115–1.722)	0.003	1.272 (1.013–1.598)	0.038	4.29	1
BUN > 21 mg/dL	1.711 (1.373–2.132)	< 0.001	1.346 (1.063–1.703)	0.013	6.12	2
AST > 32 U/L	1.384 (1.108–1.728)	0.004	1.050 (0.790–1.395)	0.739		
ALT > 59 U/L	1.585 (1.194–2.104)	0.001	1.256 (0.880–1.795)	0.210		
cTnI/T elevated	1.405 (1.123–1.757)	0.003	1.244 (0.971–1.594)	0.084		
BNP (NT–proBNP) pg/ml						
BNP 100 to < 500 (NT–proBNP 300 to < 2500)	Reference		Reference			
BNP 500 to < 1500 (NT–proBNP 2500 to < 7500)	1.679 (1.251–2.254)	0.001	1.460 (1.074–1.983)	0.016	5.85	1
BNP ≥ 1500 (NT–proBNP ≥ 7500)	2.649 (1.962–3.577)	< 0.001	1.785 (1.281–2.488)	0.001	11.69	3
QRS fraction ^a^ < 55%	1.593 (1.279–1.985)	< 0.001	1.458 (1.156–1.839)	0.001	10.15	3
Pulmonary congestion (X–ray)	1.496 (1.201–1.863)	< 0.001	0.909 (0.667–1.238)	0.544		
LVEF < 36%	1.224 (0.962–1.558)	0.100	0.955 (0.733–1.245)	0.735		
Left atrial diameter > 41 mm	1.367 (1.094–1.707)	0.006	1.232 (0.971–1.563)	0.086		
ACEI/ARB not used	1.670 (1.343–2.076)	< 0.001	1.588 (1.260–2.003)	< 0.001	15.30	4
β–blocker not used	1.357 (1.081–1.703)	0.009	1.150 (0.900–1.470)	0.264		
Tolvaptan not used	2.522 (1.201–5.293)	0.014	1.935 (0.890–4.209)	0.096		
rhBNP used	1.629 (1.160–2.288)	0.005	1.474 (1.019–2.131)	0.039	4.25	1
CCB used	1.458 (1.045–2.034)	0.026	1.230 (0.868–1.744)	0.244		
Statin used	1.291 (1.032–1.615)	0.025	1.226 (0.963–1.559)	0.098		

*BMI* body mass index, *LoHS* length of hospital stay, *SBP* systolic blood pressure, *DBP* diastolic blood pressure, *BUN* blood urea nitrogen, *LDL-C* low density lipoprotein cholesterol, *ALT* alanine aminotransferase, *AST* aspartate aminotransferase, *BNP* brain natriuretic peptide, *NT-proBNP* N-terminal pro-brain natriuretic peptide, *cTnI/T* cardiac troponin I/T, *LVEF* left ventricular ejection fraction, *NYHA* New York Heart Association, *LVEF* left ventricular ejection fraction, *ACEI* angiotensin converting enzyme inhibitor, *ARB* angiotensin receptor blocker, *CCB* calcium channel blockers, *rh-BNP* recombinant human brain natriuretic peptide, *χ*^2^*-df* Wald Chi-squared (*χ*^2^) statistic minus the degrees of freedom (df) of predictors

^a^QRS fraction is calculated by the sum of the R-wave amplitudes of the standard 12 leads (ΣR) and dividing by the sum of the absolute values of the QRS wave amplitudes of the 12 leads (ΣQRS), i.e. (ΣR/ ΣQRS) × 100%

**Fig. 1:**
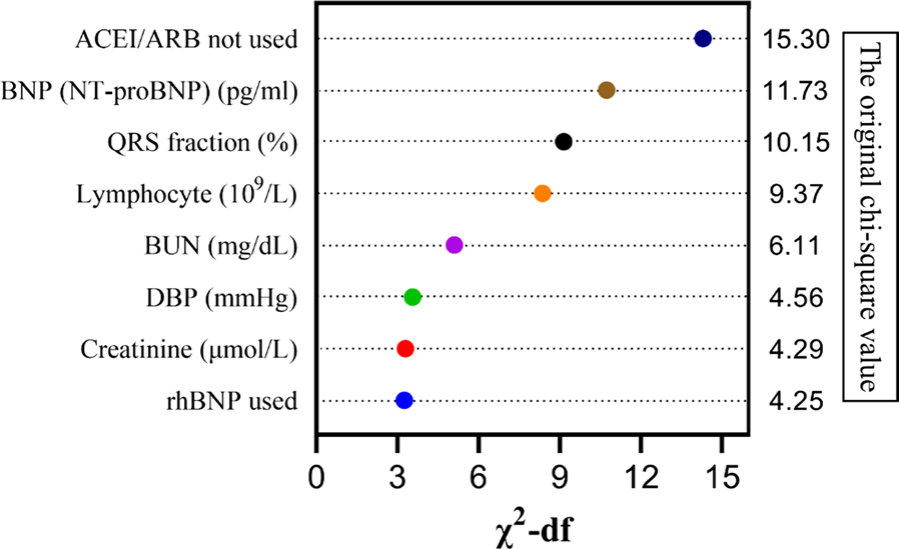
Relative importance of individual predictors within the final risk model. The relative importance of each predictor was calculated from the Wald chi-square (*χ*^2^) minus the predictor’s degrees of freedom (*df*) (*χ*^2^-*df*)

We calculated the relative importance of each independent predictor using the value of (*χ*^2^-df) (Fig. 1). The score was then assigned based on the (*χ*^2^-df) value of each independent predictor (Table 2). The new scoring system was as follows:

1 × (DBP < 80 mmHg) + 2 × (lymphocyte > 1.11 × 10^9^/L) + 1 × (creatinine > 80 μmol/L) + 2 × (BUN > 21 mg/dL) + 1 × [BNP 500 to < 1500 pg/mL (NT-proBNP 2500 to < 7500 pg/mL)] or 3 × [BNP ≥ 1500 (NT-proBNP ≥ 7500) pg/mL] + 3 × (QRS fraction of electrocardiogram < 55%) + 4 × (ACEI/ARB not used) + 1 × (rhBNP used).

### *Discrimination, calibration, and comparison of the new risk score*.

**Fig. 2:**
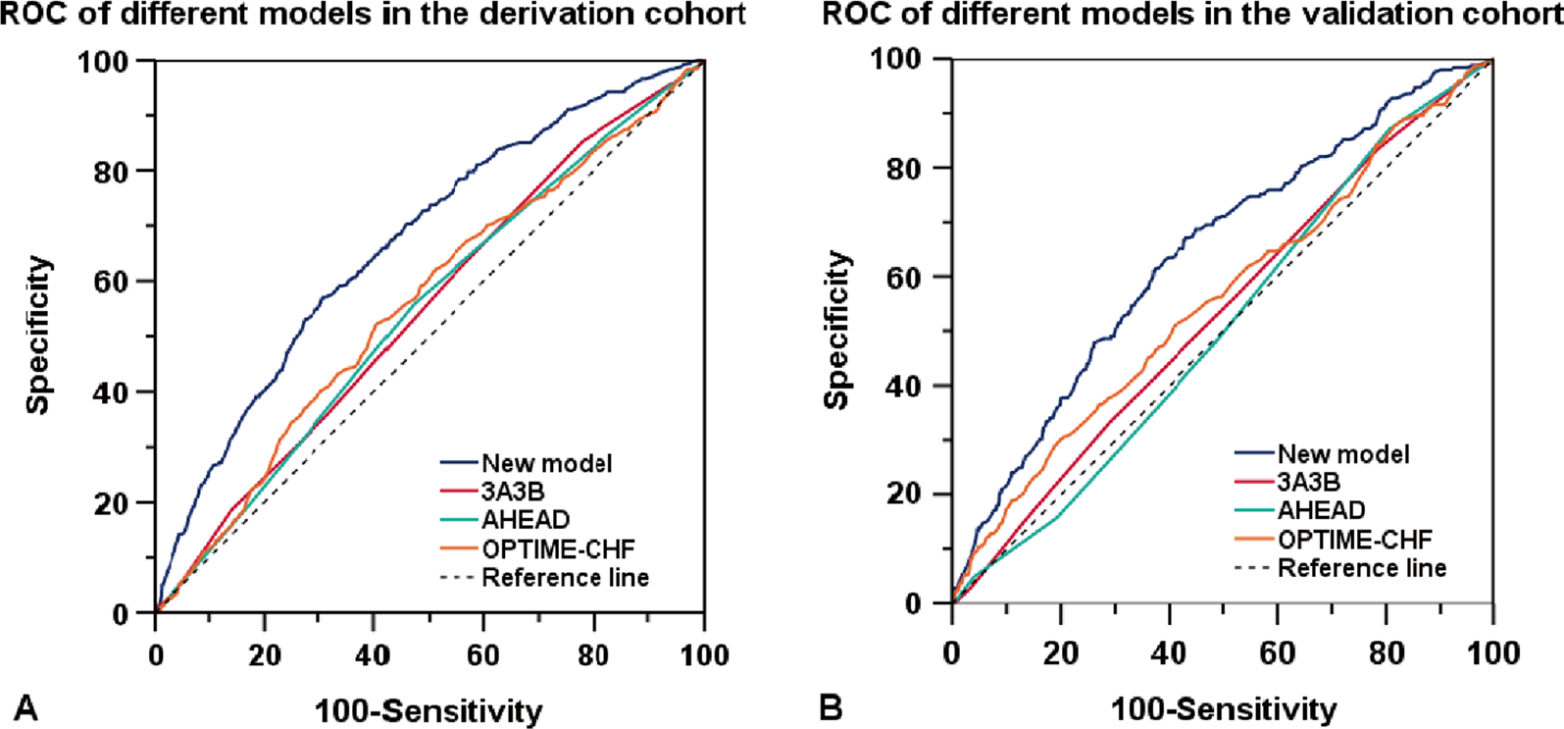
The receiver operating characteristic curve (ROC) for scoring models in the derivation and validation cohort.** A** The ROC for scoring models in the derivation cohort;** B** The ROC for scoring models in the validation cohort

The discrimination ability of the new PRS model was adequate, with an AUROC of 0.67 (95%CI 0.64–0.70) for the derivation cohort (Fig. 2A), and a good calibration (the *Hosmer–Lemeshow* test *χ*^2^ = 3.366, *P* = 0.186). The discrimination was validated by the AUROC for the validation cohort [0.65 (95% CI 0.61–0.69)] (Fig. 2B), and the calibration was likewise good (the *Hosmer–Lemeshow test χ*^2^ = 9.751, *P* = 0.283). In addition, the AUROCs of the new PRS in both the derivation and validation cohorts were the highest compared with the 3A3B, AHEAD, and OPTIME-CHF score systems (Fig. 2), of which the differences were statistically significant (*P* < 0.001; Table 3).

**Table 3:** Comparison of AUROCs between the new risk score and previous risk scores for predicting outcomes

Cohort	Comparison	Difference between AUROCs	95% CI	*Z*-statistic	*P*-value
Derivation	New risk score versus 3A3B	0.122	0.019 (0.086–0.158)	6.595	< 0.001
	New risk score versus AHEAD	0.125	0.019 (0.089–0.163)	6.515	< 0.001
	New risk score versus OPTIME–CHF	0.114	0.018 (0.078–0.150)	6.174	< 0.001
Validation	New risk score versus 3A3B	0.111	0.023 (0.066–0.156)	4.808	< 0.001
	New risk score versus AHEAD	0.136	0.025 (0.087–0.185)	5.455	< 0.001
	New risk score versus OPTIME–CHF	0.086	0.024 (0.040–0.132	3.663	< 0.001

### The risk stratification of the new risk score model in the entire cohort.

**Fig. 3:**
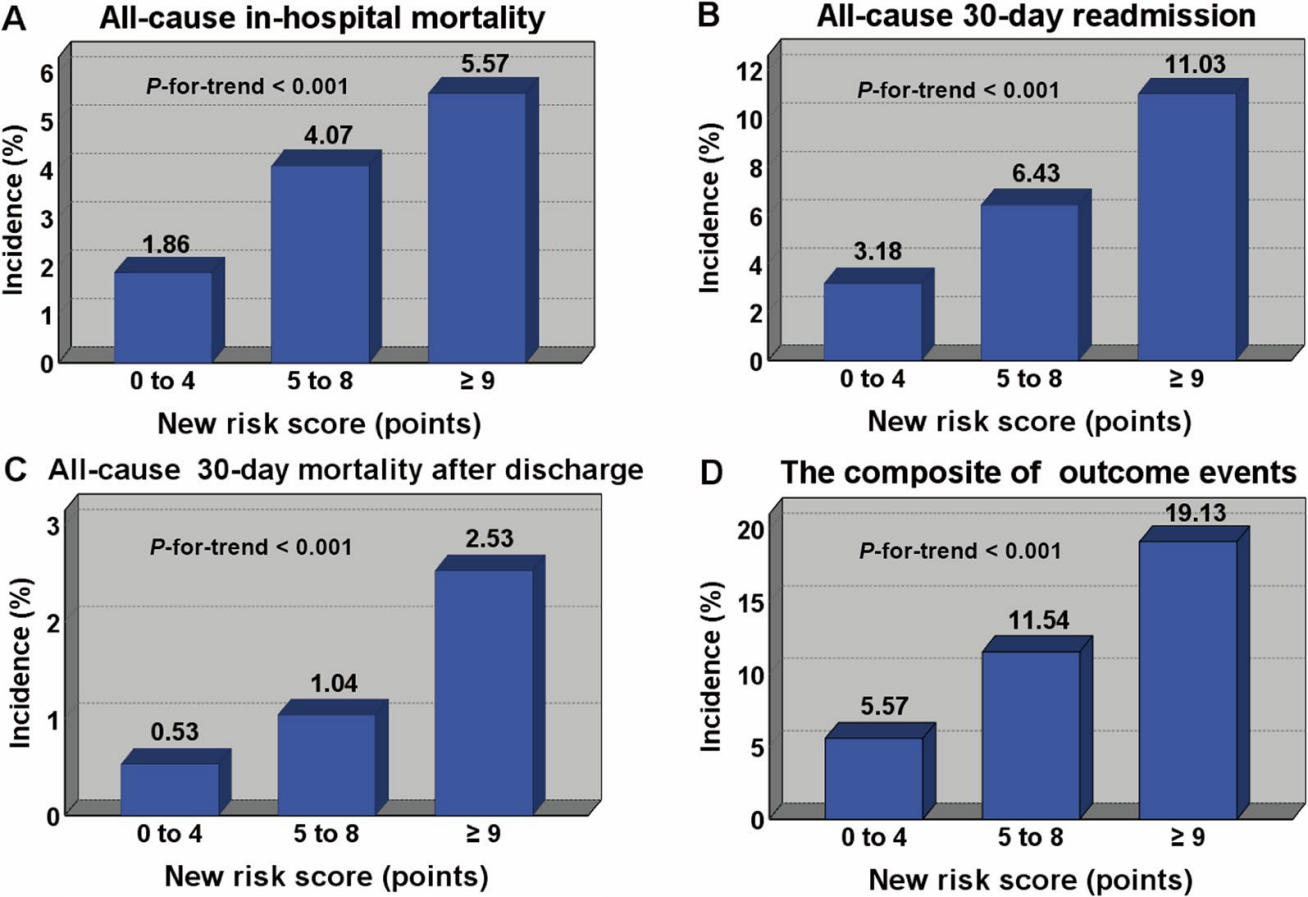
The incidence of outcome events for patients with different risk stratification in the entire cohort. The points of 0–4, 5–8, and ≥ 9 represent the low-, medium-, and high-risk stratification respectively.** A** The incidence of all-cause in-hospital mortality for patients with different risk stratification in the entire cohort;** B** The incidence of all-cause 30-day readmission for patients with different risk stratification in the entire cohort;** C** The incidence of all-cause 30-day mortality after discharge for patients with different risk stratification in the entire cohort;** D** The incidence of the compositive outcome events for patients with different risk stratification in the entire cohort

The new PRS was then used to classify patients into 3 groups: 0–4 points, low-risk group; 5–8 points, medium-risk group; ≥ 9 points, high-risk group. The incidence of in-hospital all-cause mortality in low-, medium-, and high-risk groups was 1.86% (14/754), 4.07% (74/1820), and 5.57% (110/1976), respectively (Fig. 3A). The incidence of 30-day readmission in low-, medium-, and high-risk groups was 3.18% (24/754), 6.43% (117/1820), and 11.03% (218/1976), respectively (Fig. 3B). The incidence of 30-day all-cause mortality after discharge were 0.53% (4/754), 1.04% (19/1820), and 2.53% (50/1976), respectively (Fig. 3C) and the composite of outcome events were 5.57% (42/754), 11.54% (210/1820), and 19.13% (378/1976), respectively (Fig. 3D). *Mantel–Haenszel* test revealed a linear trend between the risk stratification and the incidence of outcomes events (*χ*^2^ = 38.14, *P* < 0.001). *Pearson* correlation test indicated that the incidence of outcome events increased with the increase of the risk stratification (*R* = 0.480, *P* < 0.001).

### The new risk score model without rhBNP

Considering that rhBNP may not be widely available or used in other countries, a score without this variable was provided. After multivariate analysis, anemia, cTnI/T, BNP (NT-proBNP), QRS fraction of electrocardiogram, and ACEI/ARB were included in the final scoreing system (Table 4), which was calculated as follows:

**Table 4:** Logistic regression analysis of the composite outcomes without rhBNP in the derivation cohort

Variables	Multivariate analysis		Model selection	
	OR (95% CI)	*P-Value*	*χ*^2^-df	Score
Sex (meal)	0.787 (0.589–1.051)	0.104		
BMI < 24 kg/m^2^	0.963 (0.725–1.297)	0.793		
LoHS ≥ 10 days	0.933 (0.706–1.234)	0.629		
DBP < 80 mmHg	0.991 (0.747–1.314)	0.949		
Anemia	1.558 (1.040–2.333)	0.032	3.624	1
Red blood cells ≤ 4.15 × 10^12^/L	1.290 (0.821–2.209)	0.270		
Neutrophil > 4.37 × 10^9^/L	1.044 (0.678–1.487)	0.984		
Lymphocyte > 1.11 × 10^9^/L	1.355 (0.896–2.049)	0.150		
Platelet ≥ 155 × 10^9^/L	1.025 (0.751–1.400)	0.874		
Total cholesterol < 3.6 mmol/L	0.973 (0.656–1.444)	0.893		
LDL-C < 2.0 mmol/L	1.290 (0.853–1.950)	0.228		
Creatinine > 80 μmol/L	0.902 (0.681–1.195)	0.474		
BUN > 21 mg/dL	1.239 (0.927–1.656)	0.148		
AST > 32 U/L	1.242 (1.912–1.691)	0.169		
ALT > 59 U/L	0.740 (0.493–1.111)	0.146		
cTnI/T elevated	1.647 (1.225–2.215)	0.001	9.908	2
BNP (NT–proBNP) pg/ml				
BNP 100 to < 500 (NT-proBNP 300 to < 2500)	Reference			
BNP 500 to < 1500 (NT–proBNP 2500 to < 7500)	1.280 (1.014–1.717)	0.016	2.719	1
BNP ≥ 1500 (NT-proBNP ≥ 7500)	1.587 (1.088–2.314)	0.001	5.757	2
QRS fraction ^a^ < 55%	1.720 (1.283–2.305)	< 0.001	12.155	3
Pulmonary congestion (X-ray)	0.833 (0.572–1.214)	0.341		
LVEF < 36%	0.858 (0.615–1.196)	0.365		
Left atrial diameter > 41 mm	1.280 (0.954–1.717)	0.099		
ACEI/ARB not used	1.394 (1.046–2.857)	0.023	4.418	1
β-blocker not used	1.020 (0.750–1.386)	0.900		
Tolvaptan not used	1.763 (0.635–4.897)	0.277		
CCB used	1.019 (0.679–1.529)	0.928		
Statin used	0.820 (0.611–1.101)	0.187		

*BMI* body mass index, *LoHS* length of hospital stay, *SBP* systolic blood pressure, *DBP* diastolic blood pressure, *BUN* blood urea nitrogen, *LDL-C* low density lipoprotein cholesterol, *ALT* alanine aminotransferase, *AST* aspartate aminotransferase, *BNP* brain natriuretic peptide, *NT-proBNP* N-terminal pro-brain natriuretic peptide, *cTnI/T* cardiac troponin I/T, *LVEF* left ventricular ejection fraction, *NYHA* New York Heart Association, *LVEF* left ventricular ejection fraction *ACEI* angiotensin converting enzyme inhibitor, *ARB* angiotensin receptor blocker, *CCB* calcium channel blockers, *χ*^2^*-df* Wald Chi-squared (*χ*^2^) statistic minus the degrees of freedom (df) of predictors

^a^QRS fraction is calculated by the sum of the R-wave amplitudes of the standard 12 leads (ΣR) and dividing by the sum of the absolute values of the QRS wave amplitudes of the 12 leads (ΣQRS), i.e. (ΣR/ ΣQRS) × 100%

1 × Anemia + 2 × (cTnI/T elevated) + 1 × [BNP 500 to < 1500 pg/mL (NT-proBNP 2500 to < 7500 pg/mL)] or 2 × [BNP ≥ 1500 (NT-proBNP ≥ 7500) pg/mL] + 3 × (QRS fraction of electrocardiogram < 55%) + 1 × (ACEI/ARB not used).

The discrimination ability of the PRS model without rhBNP was suitable, whose AUROC for the derivation cohort was 0.66 (95%CI 0.63–0.70) with good calibration (the *Hosmer–Lemeshow* test *χ*^2^ = 10.270, *P* = 0.247).

## Discussions

Using the data from the Heb-ADHF registry, we externally validated 3 previously published risk models (3A3B, AHEAD, OPTIME-CHF) to predict the composite of in-hospital all-cause mortality, 30-day all-cause readmission, or 30-day all-cause mortality after discharge in patients with ADHF. The current findings revealed that all the 3 models performed poorly. We also developed a new PRS based on clinical characteristics on admission. The new scoring system comprised a combination of variables, including DBP, lymphocyte, creatinine, BUN, BNP (NT-proBNP), QRS fraction, ACEI/ARB, and rhBNP. We found that the new PRS outperformed the 3A3B, AHEAD, and OPTIME-CHF score in terms of predicting the short-term prognosis. More importantly, we first demonstrated that the QRS fraction is an independent predictor for the short-term prognosis of patients with ADHF. In addition, it was interesting that low DBP, lymphocyte count and rhBNP used were positive predictors of poor short-term outcomes in patients with ADHF in our study.

The short-term risk of death or readmission for ADHF was 13.9% in our study, which was somehow lower than the previous literature [17]. The reason may be that 18.9% of our patients with milder conditions (858 cases of cardiac function in class II). This also resulted in huge direct and indirect economic losses [1, 8, 18, 19]. Short-term prognosis assessment for patients with ADHF remains one of the major challenges for clinicians. Previous studies have demonstrated that the risk score can effectively predict the adverse outcomes of HF. Nevertheless, validation in different cohorts is a crucial step in providing evidence for the score performance, which must be considered in the context of different studies [20, 21]. The availability of the included variables determines the success of any PRS [11]. Likewise, when validating the original score in an external population, it is necessary to assess the applicability and variable availability of the original score in the existing population. We have considered employing other well-known models for the validation in our population. For example, the ADHERE model [16], only provided an inconvenient formula for calculating the log odds of mortality and was somewhat antiquated; for the EHMRG30-ST [22], we lacked data on how patients arrived at the hospital and metoprolol use prior to admission. Besides the applicability and variables availability, we chose the 3A3B, AHEAD, and OPTIME-CHF score because of their study populations involved: the OPTIME-CHF model from the United States [10], the AHEAD from Europe [12], and the 3A3B model from Asian [13]. We attempted to locate one of the existing risk models from different regions to evaluate their short-term prediction abilities for ADHF patients. Unfortunately, it did not work out. Although our endpoint-positive group had higher scores than the endpoint-negative group, the 3A3B, AHEAD, and OPTIME-CHF score performed poorly in the population of Heb-ADHF. The differences in study populations and endpoints design could explain disparity in the results. In other words, directly applying of the previously published HF risk scores to the ADHF population is debatable. This implies that the development and implementation of risk scores for different populations, as well as the variables included in these scores, should be specific and targeted.

In our study, the new PRS got a better predictive value than the 3 previous scoring systems in predicting the composite outcomes. Furthermore, our findings suggested that there was a linear correlation between the new PRS and outcome events. The higher the risk stratification, the higher the incidence of adverse outcome events (*P*-for-trend < 0.001), namely high-risk stratification increased the incidence of in-hospital all-cause mortality, 30-day all-cause readmission, 30-day all-cause mortality after discharge, or the composite outcomes. Although the discrimination (AUROC) results were not deal, it still has potential predictive value. In particular, our PRS contained some specific predictors with independent predictive value for the endpoint, such as the DBP, lymphocyte, rhBNP, and QRS fraction.

“Risk scores are multivariate predictive models in which relative weights are assigned to each variable in order to calculate the probability that a specific event (e.g. death, rehospitalization) will occur in the future” [9]. Therefore, risk variables are the basic elements of PRS, and different variables may play different roles in different PRS. In general, multiple risk variables can coexist in the same patient [23]. Anemia [12, 13], BNP or NT-proBNP [24], BUN or creatinine [25, 26], and/or ACEI/ARB use [27] have all been identified as common variables affecting the prognosis of patients with ADHF. In our new PRS model, ACEI/ARB use and high BNP (NT-proBNP) level are the two variables with the highest weights [(*χ*^2^-df) value] (Fig. 1). Additionally, more studies have suggested that low SBP is closely related to adverse outcomes in ADHF patients [28, 29]. However, studies examining the relationship between DBP and the prognosis of ADHF patients are relatively rare. Our results indicated that low DBP (< 80 mmHg), not SBP, was an independent variable of short-term adverse outcomes in patients with ADHF. Similarly, many studies have shown that hemoglobin (anemia) [12, 13], red blood cell distribution width [30], although blood cells, and/or hematocrit [31] are associated with poor prognosis in patients with ADHF, while the lymphocyte is rarely employed as a predictor. In our new PRS, higher lymphocytes count as an independent predictor could predict short-term poor prognosis for ADHF patients, which was different from the recent published research that believed patients with AHF in lower lymphocyte count had a higher risk of mortality [32]. Our study population was ADHF patients, including both new-onset AHF and decompensated CHF. The reason is not yet clear. We limited the study endpoint to short-term follow-up and the composite events, while the previous literature solely studied all-cause death with no time constraint on follow-up. In addition, lymphocytes are immunological cells, ADHF inpatients had a higher lymphocyte count [33]. Variations in the study population and design may account for the differences. More studies are needed in the future to verify this conclusion and explore the mechanisms involved.

More intriguing and meaningful, rhBNP used and low QRS fraction were also predictors of related adverse events. It is known that rhBNP is a synthetic drug frequently used in the clinical treatment of patients with ADHF and recommended by some guidelines [8, 34]. rhBNP was approved by US Food and Drug Administration in 2001, which can reduce pre-and post-load through dilated veins and arteries; it also has certain effects on promoting sodium excretion, diuresis, and inhibiting renin–angiotensin–aldosterone system and sympathetic nervous system. The drug is safe for patients with ADHF and can significantly improve hemodynamics and dyspnea-related symptoms, which is recommended for the treatment of ADHF by some HF guidelines [8, 34]. Generally speaking, rhBNP has protective effects on the heart and kidney [8, 34, 35]. Studies on patients with ADHF have demonstrated that the use of rhBNP could reduce the 30-day mortality and readmission rate [36, 37]. However, the use of rhBNP in our study, on the other hand, increased the composite outcomes. The result was somewhat unexpected. We do not believe the truth is as it appears. Although the exact proportion of rhBNP used in ADHF patients is unknown, it is unquestionably higher in severe patients. The most likely reason that treatment with rhBNP increased the composite endpoints in our study was the proportion of critical patients included in our cohort. They may need more rhBNP than those with milder conditions. This phenomenon needs further exploration. It should also be noted that rhBNP is not widely used in several other countries and is not further recommended in the latest guidelines for heart failure [38, 39]. As a result, we provided a score without rhBNP use. Although the variable composition changed slightly, its discrimination and calibration remained suitable. Moreover, the QRS fraction was always an independent predictor of the composite endpoint.

The QRS fraction included in our PRS as a novel independent predictor for the endpoints was a rather novel finding of our study. Prior published QRS scoring systems have been proved to be related to left ventricular function [40–42], but their computational complexity prevents them from convenient clinical application. Different from previous QRS scores, our novel QRS fraction is simpler and easier to achieve, which is defined as (ΣR/ΣQRS) × 100%. As described above, QRS scoring systems were associated with left ventricular function [40–42] and left ventricular function has been shown to predict the development and prognosis of heart failure [43]. This may be the reason why the QRS fraction can predict outcomes of our cohorts. To the best of our knowledge, this is the first study to use this method to evaluate the prognosis in patients with ADHF. After correction for confounding variables, the low QRS fraction remained an independent predictor of the composite of in-hospital all-cause mortality, 30-day all-cause readmission, or 30-day all-cause mortality after discharge for patients with ADHF. Excitingly, our results indicate that a low QRS fraction (< 55%) has a higher weight in our new PRS (Fig. 1). The new PRS outperformed the 3A3B, AHEAD and OPTIME-CHF scores, which was largely due to the introduction of the QRS score. We believe the newly found QRS fraction is a simple-to-obtain and practical parameter to employ in daily clinical practice. This is an issue that needs to be further addressed and we believe that combining the QRS fraction in future PRS will demonstrate its real value in predicting the short-term prognosis of ADHF patients.

In fact, using PRSs for ADHF in clinical practice is far from enough. A single PRS is unlikely to apply to all ADHF populations. However, if effective and simple PRSs are actively applied in daily clinical work and incorporated into electronic health records like the application of CHA_2_DS_2_-VASc score in atrial fibrillation [44] or GRACE score in acute myocardial infarction [45], we believe that patients with ADHF will receive better treatment and have a better prognosis. To achieve this goal, clinicians and researchers still have a long way to go.

## Study limitations

There exist several limitations in the present study. First, patients' socio-economic background may strongly influence treatment. Some diagnostic and therapeutic approaches such as the detection of interleukin-1 receptor-like 1 (ST2), the use of angiotensin receptor-neprilysin inhibitor (ARNI), and the use of implantable cardioverter defibrillator (ICD) or cardiac resynchronization therapy-defibrillator (CRT-D), etc. were less common in the our study population, which may have influenced the inclusion of valuable variables and the discrimination for our new PRS. In the future, it will be necessary to include more updated variables to improve predictive accuracy. Second, because our study was conducted in multi-centers, some only tested BNP, while others only tested NT-proBNP, which may have a certain impact on the results. Third, other than the 3A3B, AHEAD and OPTIME-CHF scores, we have considered comparing the predictive accuracy of the new PRS with other scores in our population. Unfortunately, we failed since our cohort lacked key variables contained in other scores. Fourth, the clinical data were collected for patients on admission without considering pre-hospital management and there was a small amount of data missing, which could have influenced the results. Besides, our PRS still needs external validation.

## Conclusions

Collectively, the present study validated 3A3B, AHEAD, and OPTIME-CHF score in ADHF patients from the Heb-ADHF registry, but their discriminations were all poor to predict the composite of in-hospital all-cause mortality, 30-day all-cause readmission, or 30-day all-cause mortality after discharge. We proposed a new short-term PRS (including DBP, lymphocyte, creatinine, BUN, BNP (NT-proBNP), QRS fraction, ACEI/ARB, and rhBNP) that showed moderate predictive capability with the larger AUROC than the 3A3B, AHEAD, and OPTIME-CHF scores. The newly found QRS fraction is an easy to obtain and practical parameter employed in daily clinical practice. When the risk score reaches ≥ 9 points, special attention must be paid and clinical decision-making must be formulated and adjusted more actively. More new variables and larger multicenter studies are required to make our PRS become a practical and reliable tool for evaluating the short-term adverse events for ADHF patients.

## Data Availability

The authors confirm that the data supporting the findings of this study are available within the article. The datasets used and analysed during the current study are available from the corresponding author on reasonable request.
